# Supplementary material for: Machinery and Developmental Role of Retinoic Acid Signaling in Echinoderms
Source: Cells. 2022 Feb 2;11(3):523. doi: 10.3390/cells11030523 (PMC8834484; doi:10.3390/cells11030523)
Supplement: Supplementary file 1 [file cells-11-00523-s001.zip › Supplementary file/Fig. S1.pdf]

```
Human_Hs_aldh1a1         1 M-SSSGT----------PD--------------LPVLLTDLKIQYTKIFINNEWHDSVSGKKFPVFNPATEEELCQ  51
Human_Hs_aldh1a2         1 M-TSSKIEMPGEVKADPAALMA-------SLHLLPSPTPNLEIKYTKIFINNEWQNSESGRVFPVYNPATGEQVCE  68
Human_Hs_aldh1a3         1 MATANGAVENGQPDRKPPA--------------LPRPIRNLEVKFTKIFINNEWHESKSGKKFATCNPSTREQICE  62
SeaLily_Mr_aldh1-a       1 M--------NLQLVFSRPFLLSVNRGFFVHSYSMSQLNSAPEVKFTQLFINNEFVNSVSGKTFPTLNPCTGEKICD  68
Starfish_Pp_aldh1a-a     1 -------------------------------------MAPPQVKYTQLFINNEFVNSVSGKTFPTINPQTGEKICD  39
Starfish_Pp_aldh1a-b       ----------------------------------------------------------------------------
Starfish_Pp_aldh1a-c     1 -------------------------------------MAPVEVKYTQIFINNEFVNSVSGKTFPTLNPATGKKTCD  39
BrittleStar_Af_aldh1a    1 -------------------------------------MAAPEVRFTQIFINNEFVNSVSGKTFPCINPINQKKVTD  39

Human_Hs_aldh1a1        52 VEEGDKEDVDKAVKAARQAFQIGSPWRTMDASERGRLLYKLADLIERDRLLLATMESMNGGKLYSNAYLNDLAGCI 127
Human_Hs_aldh1a2        69 VQEADKADIDKAVQAARLAFSLGSVWRRMDASERGRLLDKLADLVERDRAVLATMESLNGGKPFLQAFYVDLQGVI 144
Human_Hs_aldh1a3        63 VEEGDKPDVDKAVEAAQVAFQRGSPWRRLDALSRGRLLHQLADLVERDRATLAALETMDTGKPFLHAFFIDLEGCI 138
SeaLily_Mr_aldh1-a      69 VQEGDKADVDLAVKAAREAFKLGSPWRRLDPTKRAKHMTKLAELLEQNKDQLSALETLDNGMPYFESQ-MWVDSFV 143
Starfish_Pp_aldh1a-a    40 IQEGDKADVDLAVKAAREAVKLGSPWRTMDAQDRGKLMQKLADLFERDAEHIASLDTLDNGTLYTTMY-GCVQGSV 114
Starfish_Pp_aldh1a-b     1 ----------------------------MDASKRALYLLKLAELLKRDKEYLASLETLDNGLIIAGSR-SCVDRAV  47
Starfish_Pp_aldh1a-c    40 VQEGDKADADKAVRAAQDAFKLGSAWRRMDATGRARLLNKLADLIERDREYLVNLETMDNGMTVMNAT-GSVMGCA 114
BrittleStar_Af_aldh1a   40 IQEGDKADVDKAVQAARDAFKLGSPWRRMDAAARGRLLMKLADLVERDADYISKLETSDNGMLVKNAA-GCVEMAV 114

Human_Hs_aldh1a1       128 KTLRYCAGWADKIQGRTIPIDGNFFTYTRHEPIGVCGQIIPWNFPLVMLIWKIGPALSCGNTVVVKPAEQTPLTAL 203
Human_Hs_aldh1a2       145 KTFRYYAGWADKIHGMTIPVDGDYFTFTRHEPIGVCGQIIPWNFPLLMFAWKIAPALCCGNTVVIKPAEQTPLSAL 220
Human_Hs_aldh1a3       139 RTLRYFAGWADKIQGKTIPTDDNVVCFTRHEPIGVCGAITPWNFPLLMLVWKLAPALCCGNTMVLKPAEQTPLTAL 214
SeaLily_Mr_aldh1-a     144 NTLTYFAGWCDKVHGKTIPIDGDYFCYTKHEPVGVCGAIIPWNYPMDMLGWKVGPALACGNTMVIKPAEQTPLTAL 219
Starfish_Pp_aldh1a-a   115 EVLRYFAGWADKIHGKTIPIKGDFFCYTRHEPVGVCGAIIPWNFPTEMLSWKLGPALCCGNSLIIKPAEQTPLSAL 190
Starfish_Pp_aldh1a-b    48 GLLHYYAGWADKITGKTIPIDGSFFCYTRYEPKGVVAAVTPWNAPIVVTTRKMATALACGNTVVLKPAEQTPLTAL 123
Starfish_Pp_aldh1a-c   115 KFLRYGAGYADKLHGKVVPLDGDYFCYSRYEPIGVVAAIIPWNFPCLLTGAKLSCALTVGNTLVIKPAEQSPLTAL 190
BrittleStar_Af_aldh1a  115 SVLRYYGGYADKIHGKTIPIDGDFFCYTRHEPIGVCGAIIPWNFPIEMASWKMGPALACGNTMVIKPAEQTPLTAL 190

Human_Hs_aldh1a1       204 HVASLIKEAGFPPGVVNIVPGYGPTAGAAISSHMDIDKVAFTGSTEVGKLIKEAAGKSNLKRVTLELGGKSPCIVL 279
Human_Hs_aldh1a2       221 YMGALIKEAGFPPGVINILPGYGPTAGAAIASHIGIDKIAFTGSTEVGKLIQEAAGRSNLKRVTLELGGKSPNIIF 296
Human_Hs_aldh1a3       215 YLGSLIKEAGFPPGVVNIVPGFGPTVGAAISSHPQINKIAFTGSTEVGKLVKEAASRSNLKRVTLELGGKNPCIVC 290
SeaLily_Mr_aldh1-a     220 HIASLIKEAGFPPGVINIIPGYGPTAGAAISEHMDVDKVAFTGSTEVGRLIQQAAGKSNLKRVALELGGKSPNIVF 295
Starfish_Pp_aldh1a-a   191 HIASLIKEAGFPAGVVNIIPGYGPTAGAAISEHMDVDKVAFTGSTEIGRLIQAASGTSNLKRVSLEMGGKSPNIIF 266
Starfish_Pp_aldh1a-b   124 YLASLIKEAGFPPGVVNVVPGYGPTAGAALSEHMDVDVITFTGSVEVGKLIQQASGKSNLKHVCLELGGKSPNVVF 199
Starfish_Pp_aldh1a-c   191 YIASLAKEAGFPPGVINVVPGYGPTAGAALTGSMDVDMVTFTGSTEVGRIIQRAGADSNLKKIHLELGGKSPNVVF 266
BrittleStar_Af_aldh1a  191 YLASLVKEAGFPPGVVNFVPGYGPTAGAAISEHMNVDKVAFTGSTEIGKIIQQAAGKSNLKRVSLEMGGKSPNIVF 266

Human_Hs_aldh1a1       280 ADADLDNAVEFAHHGVFYHQGQCCIAASRIFVEESIYDEFVRRSVERAKKYILGNPLTPGVTQGPQIDKEQYDKIL 355
Human_Hs_aldh1a2       297 ADADLDYAVEQAHQGVFFNQGQCCTAGSRIFVEESIYEEFVRRSVERAKRRVVGSPFDPTTEQGPQIDKKQYNKIL 372
Human_Hs_aldh1a3       291 ADADLDLAVECAHQGVFFNQGQCCTAASRVFVEEQVYSEFVRRSVEYAKKRPVGDPFDVKTEQGPQIDQKQFDKIL 366
SeaLily_Mr_aldh1-a     296 ADSDLDFAVDEAHEAVMCNEGQCCSAGSRTFVQEGIYDEFVKKSIEMAKARVIGDPYVEGTQSGPQIDEEQFTKVL 371
Starfish_Pp_aldh1a-a   267 ADADLDYAVEESHEAIFFNMGECCSAGSRTFVQEGIYDEFVKKSVERAKRRVVGDPFEEKTESGPQIDQDQMDKIL 342
Starfish_Pp_aldh1a-b   200 ADADLDYAVETSHRALFAHSGQICIAGSRTFVQEDIYDEFVKKSTERAKKRVVGDPYNDKSESGPVIDEGQVTRIL 275
Starfish_Pp_aldh1a-c   267 ADCDLDYAVEMSHYGVFLHSGQVCCAGTRTFVQEDIYDEFVRKSKERAEKRVVGDPYDVKTEGGPQIDQDQLDKIL 342
BrittleStar_Af_aldh1a  267 ADCDLDYAVEEAHEALFFNMGECCCAGSRTYVQEGIYDEFVKKSVERAKTRVVGNPYDDKTENGPQIDQEQFDKIM 342

Human_Hs_aldh1a1       356 DLIESGKKEGAKLECGGGPWGNKGYFVQPTVFSNVTDEMRIAKEEIFGPVQQIMKFKSLDDVIKRANNTFYGLSAG 431
Human_Hs_aldh1a2       373 ELIQSGVAEGAKLECGGKGLGRKGFFIEPTVFSNVTDDMRIAKEEIFGPVQEILRFKTMDEVIERANNSDFGLVAA 448
Human_Hs_aldh1a3       367 ELIESGKKEGAKLECGGSAMEDKGLFIKPTVFSEVTDNMRIAKEEIFGPVQPILKFKSIEEVIKRANSTDYGLTAA 442
SeaLily_Mr_aldh1-a     372 EKIKSGKNEGATLGCGGSRHGDKGFFLESTVFSDVSDEMSIAQEEIFGPVQVILKFKTIEEVIERAHKTHYGLAGA 447
Starfish_Pp_aldh1a-a   343 SYVEIGKKEGAKLECGGQRIGDKGYFVQSTVFSNVTNEMRVAQEEIFGPVQLLIKFKTLDEVLEKANNTQYGLAGG 418
Starfish_Pp_aldh1a-b   276 GMIESGKEEGAKLQCGGIRSDRKGNFIESTVFSDVTDDMEIAREEIFGPVQQILKFKTLDEVIERANNTSYGLAAA 351
Starfish_Pp_aldh1a-c   343 ELIESGKKEGAKLMCGGQRKGDCGYFVESTVFSDVTDDMRIAKEEIFGPVQQILKFKTIEEVVERANATTYGLAGA 418
BrittleStar_Af_aldh1a  343 ELIEAGKKEGAKLQCGGKQ--KDGWFIESTVFSDVTDNMRIAREEIFGPVQTIFKFKTLEEVIERANDTQYGLAAA 416

Human_Hs_aldh1a1       432 VFTKDIDKAITISSALQAGTVWVNCYGVVSAQCPFGGFKMSGNGRELGEYGFHEYTEVKTVTVKISQKNS       501
Human_Hs_aldh1a2       449 VFTNDINKALTVSSAMQAGTVWINCYNALNAQSPFGGFKMSGNGREMGEFGLREYSEVKTVTVKIPQKNS       518
Human_Hs_aldh1a3       443 VFTKNLDKALKLASALESGTVWINCYNALYAQAPFGGFKMSGNGRELGEYALAEYTEVKTVTIKLGDKNP       512
SeaLily_Mr_aldh1-a     448 VFTKDIDTAMTVAHSLSAGTVWVNCYNVGGPQTPFGGYKQSGVGRDLGEDSLKEYYEVKTVIIKVPQKNS       517
Starfish_Pp_aldh1a-a   419 VFTKDIDKALTIANSLQAGTITVNNYSGGGANAPFGGYKMSGIGRDLGEDALHEYYQVKTVIVKVPVKNS       488
Starfish_Pp_aldh1a-b   352 VITKDIDKALTMANSVRAGVVWVNTYSVLDPSAPFGGYKMSGIGREGGEESLLEFSEIKTVVIKIPQKNS       421
Starfish_Pp_aldh1a-c   419 VFTRDIDKALTVANTIRAGLIWVNNYGLFSPMIPFGGYKMSGVGREGGEDGFKEYCEVKTVVIKVPQKNS       488
BrittleStar_Af_aldh1a  417 VFSKDIDKALTISNSVRAGTVWINNYNMSSAQTPFGGYKMSGNGRELGEAALHEYYEVKTVIVKIPQKNS       486
```

Homotetrameric interface

NAD binding site

Catalytic residues

**Figure S1**. The alignment of echinoderm and human *aldh1a* genes.

Functional domain residues of the Human *aldh1a1* gene were predicted using the NCBI conserved domains search (https://www.ncbi.nlm.nih.gov/Structure/cdd/wrpsb.cgi). Green, red and blue shade respecitively indicates the alignment of homotetrameric interface, the NAD binding site and the catalytic residues. The percentage of the identical over aligned residues across human and each echinoderm *aldh1a* genes is indicated below. Homotetrameric interface: Sea lily; 50/61 (82.0%), Starfish 1; 48/61 (78.7%), Starfish 2; 41/59 (69.5%), Starfish 3; 38/61 (62.3%) and Brittle star; 49/61 (80.3%). NAD binding site: Sea lily; 18/18 (100%), Starfish 1; 16/18 (88.9%), Starfish 2; 16/18 (88.9%), Starfish 3; 18/18 (100%) and Brittle star; 16/18 (88.9%). Catalytic residues: Sea lily; 4/4 (100%), Starfish 1; 4/4 (100%), Starfish 2; 4/4 (100%), Starfish 3; 4/4 (100%) and Brittle star; 4/4 (100%).
