# Supplementary material for: Machinery and Developmental Role of Retinoic Acid Signaling in Echinoderms
Source: Cells. 2022 Feb 2;11(3):523. doi: 10.3390/cells11030523 (PMC8834484; doi:10.3390/cells11030523)
Supplement: Supplementary file 1 [file Fig. S2.pdf › Supplementary file/Fig. S2.pdf]

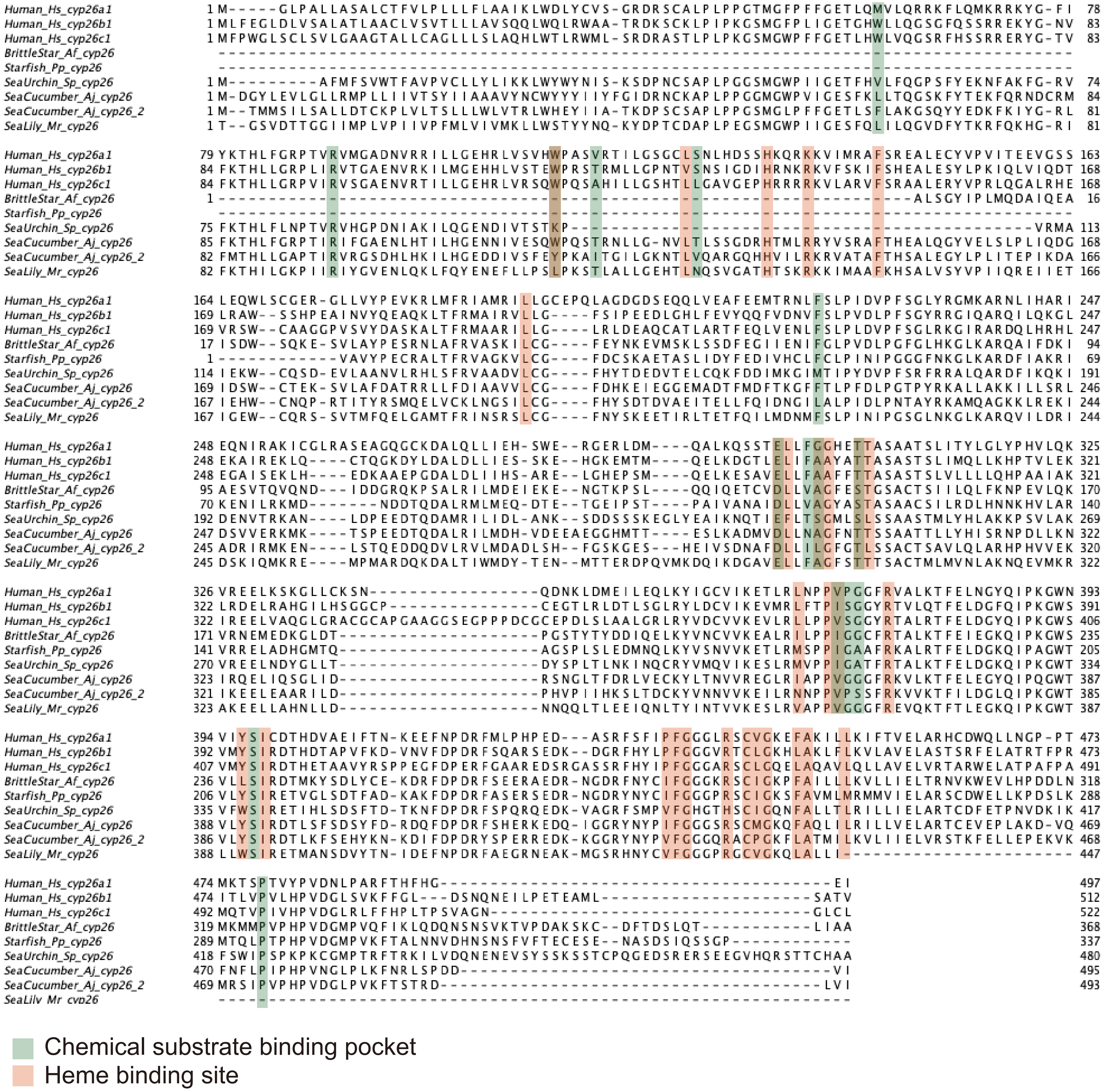

Figure S2. The alignment of echinoderm and human *cyp26* genes.
Functional domain residues of the Human *cyp26* gene were predicted using the NCBI conserved domains search (https://www.ncbi.nlm.nih.gov/Structure/cdd/wrpsb.cgi). Green and red shade respecitively indicates the alignment of the chemical substrate binding pocket and the heme binding site. The percentage of the identical over aligned residues across human and each echinoderm *cyp26* genes is indicated below. The chemical substrate binding pocket: Brittle star; 5/9 (55.6%), Starfish; 5/9 (55.6%), Sea urchin; 5/12 (41.7%), Sea cucumber 1; 9/14 (64.3%), Sea cucumber 2; 5/14 (35.7%) and Sea lily; 9/13 (69.2%). Heme binding site: Brittle star; 17/23 (73.9%), Starfish; 17/23 (73.9%), Sea urchin; 16/24 (66.7%), Sea cucumber 1; 22/28 (78.6%), Sea cucumber 2; 20/28 (71.4%) and Sea lily; 21/27 (77.8%).
