# Supplementary material for: Machinery and Developmental Role of Retinoic Acid Signaling in Echinoderms
Source: Cells. 2022 Feb 2;11(3):523. doi: 10.3390/cells11030523 (PMC8834484; doi:10.3390/cells11030523)
Supplement: Supplementary file 1 [file Fig. S3.pdf › Supplementary file/Fig. S3.pdf]

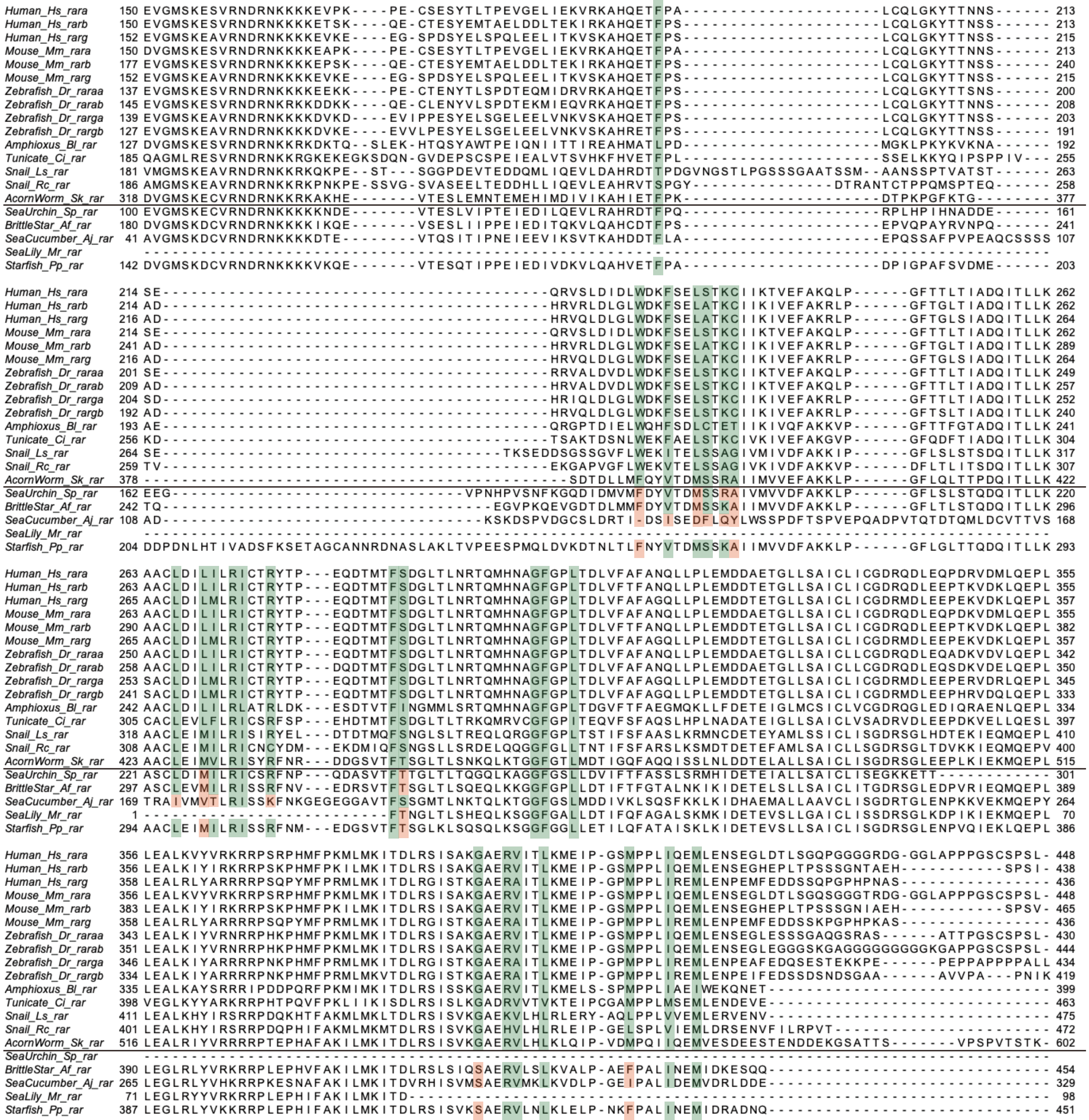

Figure S3. The alignment of echinoderm and other animal *rar* genes.
25 residues which are shaded in green color formed the ligand binding pocket in the vertebrate RAR [19]. Red sahdes indicate the mismach residues across human and echinoderm RARs. The percentage of the identical over aligned residues across human and each echinoderm *rar* genes is indicated below: Sea urchin; 12/19 (63.2%), Brittle star; 18/25 (72.0%), Sea cucumber; 13/25 (52.0 %), Sea lily; 4/5 (80.0%) and Starfish; 19/25 (76.0 %).
